# Comparison of Two Entry Methods for Laparoscopic Port Entry: Technical Point of View

**DOI:** 10.1155/2012/305428

**Published:** 2012-06-13

**Authors:** Adriana Toro, Maurizio Mannino, Giovanni Cappello, Andrea Di Stefano, Isidoro Di Carlo

**Affiliations:** Department of Surgical Sciences, Organ Transplantation, and Advanced Technologies, University of Catania, Cannizzaro Hospital, Via Messina 829, 95126 Catania, Italy

## Abstract

Laparoscopic entry is a blind procedure and it often represents a problem for all the related complications. In the last three decades, rapid advances in laparoscopic surgery have made it an invaluable part of general surgery, but there remains no clear consensus on an optimal method of entry into the peritoneal cavity. The aim of this paper is to focus on the evolution of two used methods of entry into the peritoneal cavity in laparoscopic surgery.

## 1. Introduction

Access into the abdomen is the one challenge of laparoscopy that is particular to the insertion of surgical instruments through small incisions. Laparoscopy is currently widely used in the practice of medicine, for both diagnostic and therapeutic purposes. The minimally invasive approach has become the method of choice for treating most benign abdominal diseases that require surgery. However, it is obvious that laparoscopic procedures are not risk free. Laparoscopic entry is a blind procedure, and it represents a problem for all the related complications. Complications arising from laparoscopic surgery are rare and commonly occur when attempting to gain access to the peritoneal cavity [1]. Creation of the pneumoperitoneum is the first and most critical step of a laparoscopic procedure because that access is associated with injuries to the gastrointestinal tract and major blood vessels and at least 50% of these major complications occurs prior to commencement of the intended surgery. This complication rate has remained the same during the past 25 years [2]. 

The number of vascular injuries in laparoscopy is 2 in 10.000 procedures and a serious complication associated with mortality occurs in 3.3 per 100.000 [3]. Finding a safe entry technique is a priority not only for the life of the patients but also for the increasing rate. In the last three decades, rapid advances in laparoscopic surgery have made it an invaluable part of general surgery, but there remains no clear consensus as an on optimal method of entry into the peritoneal cavity. 

There are two methods for creating a pneumoperitoneum, the closed technique and the open technique. Although there is no consensus regarding the best method of gaining access to the peritoneal cavity to create a pneumoperitoneum, the Veress needle insertion is the most frequently used technique. 

Aim of this paper is to focus on the evolution of the most used methods of entry into the peritoneal cavity in laparoscopic surgery with particular attention to patients submitted to previous surgery without comorbidities. 

## 2. Most Used Methods of Entry

Classic closed technique (Verres needle) [4] and open classic technique (Hasson technique) [5] are the common most procedures used in laparoscopy to entry into the peritoneal cavity. 

### 2.1. Verres Needle

The Verres needle is the oldest method, developed by Dr. Verres in 1938 and it is the most used technique especially in gynecological procedures. The users of this technique describe this entry as easy and quick. 

Commercially available Verres needles vary from 12 to 15 cm in length, with an external diameter of 2 mm. A bezel-shaped tip enables the needle to pierce the tissues of the abdominal wall. Upon entering the peritoneal cavity, the resistance generated from the abdominal wall is overcome, which permits the exposure of the interior needle with its blunt atraumatic mandril [6]. This system affords a degree of safety and efficacy, making the puncture of the peritoneal cavity with a Verres needle an easy, fast, and effective technique. Once the peritoneal cavity is inflated by this technique, the first trocar can be inserted without problems, minimizing intraoperative gas leakage and saving surgical time.

Nevertheless, despite this safety device, incorrect insufflations occur. Injuries to major vessels are the leading intraoperative cause of death associated with laparoscopic procedures [7]. The classic location of the Verres needle puncture is the midline of the abdomen near the umbilical scar. Due to the short distance between the anterior abdominal wall and the retroperitoneal vascular structures in this region, less than two centimetres in thin people, puncture poses risks of injury to these large vessels [8]. The abdominal aorta, the inferior vena cava, and the common iliac vessels are especially vulnerable to lesions during puncture with the Verres needle in proximity of the umbilical scar.

The needle relies on the ability of the blunt outer sheath to retract while passing through tissue and to spring forward in order to cover the sharp needle tip when tissue resistance diminishes. The length of the Verres needle that should be inserted in the abdominal cavity is not specified in any scientific report. The use of a click sound associated with the springing forward of the blunt stylet is recommended to determine when to stop advancing the needle. Unfortunately the quality of the sound is not always reliable because it depends on many factors including ambient noise and the extent of recoil in the needle spring function. The judgement is subjective and it is neither quantifiable nor taught in training. There are two important factors in the insertion of a Verres needle. First the insertion should be not excessive to avoid the risk of vascular injury. Second it should be adequate to avoid extraperitoneal insufflation, because this will lead to failure of the pneumoperitoneum with an associated operative difficulty due to inappropriate distension of the anterior abdominal wall and postoperative pain.

Tests can be performed before insufflation to verify whether the Verres needle is correctly positioned, thus avoiding injury. 

Traditional texts recommend an insertion angle of 45° from horizontal in patients with a body mass index smaller than 30 kg/m^2^ to avoid a vascular injury. Some Authors report not having a problem with a vertical orientation of the Vessel needle, provided that the umbilicus is significantly elevated and the needle is only inserted a distance of approximately 2 to 3 cm or until a negative pressure is encountered [9]. 

Different methods are reported in literature to improve the safety, for example the palpation of aorta [10], the angling of the needle [11], the saline drop test [12], the spinal needle test [13], imaging (CT and MRI), and the direct measuring of the distance [14]. 

One of the major challenge in port entry is the small bowel lesions. Usually adhesions of small bowel can be detected by ultrasound. Characteristics of preoperative abdominal ultrasound in predicting infraumbilical adhesions have been determined in a study. The results were that prevalence of infraumbilical bowel adhesions was 12%. A visceral slide threshold <1 cm to predict adhesions had sensitivity = 86%, specificity = 91%, positive predictive value = 55%, and negative predictive value = 98%. Measuring visceral slide improves preoperative prediction of both presence and absence of bowel adhesions in patients with previous abdominal operations of infections; this technique may assist in avoiding iatrogenic bowel injury [15].

This study has been integrated by test with technique of periumbilical ultrasound-guided saline infusion (PUGSI). The technique as described below is one of the most used. The presence or absence of visceral slide is demonstrated using exaggerated inspiration/expiration after intubation.

Visceral slide in this investigation is defined in accordance with Kodama's original definition as the longitudinal distance the intestines or omentum travels as visualized by ultrasound during an exaggerated inspiration/expiration cycle [16].

An abnormal test for the visceral slide is considered a movement of viscera less than 1 cm. A normal test for the viscera is movement equal to or greater than 1 cm. Immediately after the visceral slide measurement, the PUGSI test is conducted. A sterile 19-gauge spinal needle on a syringe is advanced through the skin and subcutaneous layers under ultrasound guidance at a 90° angle. Once beyond the peritoneum, 8–10 mL of sterile normal saline is injected under real-time ultrasound visualization. A normal test for PUGSI is defined as dispersion of the infused saline without fluid loculation. The formation of a fluid pocket or localization indicated an abnormal test and suggests the presence of obliterating subumbilical adhesions. An inconclusive test is defined as one in which the physician is unable to definitively determine the normality or abnormality of the test. In case of difficulties to well understand the PUGSI results the physician uses his clinical judgment to make decisions related to the best method of entry. The PUGSI test was able to detect all case of obliterating subumbilical adhesions, demonstrating sensitivity, and specificity of 100%. Use of both tests preoperatively appears to be helpful in identifying patients at risk for visceral injury during laparoscopic surgery [17].

Patients with previous abdominal surgery are more prone to visceral injury caused by the Verres needle. This is due to peritoneal adhesions, which typically grow where the incision of the parietal peritoneum was made [18]. Autopsy studies have found adhesions in 74% to 95% of patients with previous abdominal surgery. Midline incisions greatly increase the risk of adhesions in the umbilical region. Even incisions made away from the umbilicus may lead to adhesion formation in the periumbilical region. On the other hand, insertion of the Verres needle into the left hypochondrium has been reported as safe, with reduced risk of iatrogenic injury [19]. The stomach is immediately below the anterior abdominal wall at the site where the left hypochondrium puncture is made. If the stomach is accidentally perforated, its contents will not necessarily leak. This is due to the protection provided by the three layers of gastric muscle, which tend to close the puncture.

A stomach perforation is easy to diagnose upon initial inspection of the peritoneal cavity and can be repaired by laparoscopic suture.

Recently another procedure has been developed to calculate the length of the needle in order to avoid vascular lesions when it is introduced into the peritoneal cavity. A monogram has been developed to determine the length of the Verres needle that could be safely introduced to achieve the pneumoperitoneum reducing or eliminating the risk of vascular injuries of the retroperitoneal vessels during laparoscopic entry. Axial images of the magnetic resonance imaging (MRI) have been used to measure the vertical distance between umbilicus and retroperitoneal vessels to develop this procedure [20].

Two vertical measurements have been calculated. The first is the vertical distance from the skin at the pit of umbilicus to rectus sheath (STP). The second measurement is the vertical distance from the skin at the pit of umbilicus to the anterior surface of retroperitoneal vessel on the image (STR). 

The abdominal cavity depth has been defined as the vertical distance from rectus sheath at the level of umbilicus to the anterior surface of retroperitoneal vessels (the difference between the STP and STR). Two independent observers have confirmed all measurements [20]. 

Using the mean regression line for STP (skin to peritoneum) a safe insertion distance has been identified and a monogram has been developed which can be used to predict objectively the depth of the peritoneal cavity.

Specific measures for the correct insertion and for the reduction of the risk of injury of obese and thin patients have to be improved. The Verres needle insertion at 45° from the umbilicus means that needle has to traverse a distance of 12–16 cm, which increases the risk of extraperitoneal insufflation. This method using this nomogram gives a measure of the safe distance in obese patients for vertical insertion and it improves the success and safety of the umbilical insufflation. The nomogram is also helpful in thin patients as this reminds surgeon to be aware of the very short distance (+2 cm) between umbilical skin and major vessels.

The only controversy of this technique is the need of MRI for each patients, and its related time and cost.

The safe laparoscopic entry guidance should be disseminated widely but not necessarily negate the risk of laparoscopic entry-related injury, nor would it protect the clinician against any ruling of negligence should a complication occur. Unless practice concurs with recommended guidance, patients undergoing laparoscopy will be exposed to increased unnecessary operative risk [21].

### 2.2. Hasson Technique

Hasson first described open laparoscopy in 1971 and it remains the favourite entry method for many laparoscopic surgeons [5].

Some authors believe that trocar injuries to abdominal viscera occur (a) when the viscera are unusually close to the point of trocar insertion or (b) where the trocar penetrates too far into the abdominal cavity as it is inserted. The former can be anticipated when the patient has undergone a surgery previously [2]. 

In this case for avoiding visceral injury can be used the open Hasson technique or if the closed technique is used to place the first trocar at a site remote from the previous incision.The concept in the open technique is to create a tiny incision, directly incise the layers of the abdominal wall, directly cut the peritoneum and enter the abdomen. Since gas can escape around the incision, an olive is placed over the end of the trocar to occlude the incision, and sutures are placed on the abdominal fascia and attached to the cannula [2].

The benefits of this method of entry are the prevention of bowel injury caused of blind puncture with a needle and subsequent trocar, gas embolism, avoid preperitoneal insufflation and to have certainty of establishing a pneumoperitoneum, a very low incidence of vascular injuries, and furthermore a correct anatomical repair of the abdominal wall incision.

Reasons for limiting the use of the open technique include greater time needed for performance, difficulty with the technique, obese patients, and difficulty in maintenance of the pneumoperitoneum.

Open procedures are commonly employed for high-risk patients, like those with a previous abdominal surgery, in particular midline incisions or obesity [22]. An additional factor might be the higher incidence of complications early on in the surgical learning curve. Safe access depends critically on adhering to well-recognized principles of trocar insertion, knowledge of abdominal anatomy, and recognition of the hazards imposed by previous surgery. 

Widespread use of this technique has been limited to patients with previous lower abdominal surgery, pregnant women, children, and very thin patients where little space exists between the abdominal wall and the spine [2].

In experienced hands the open technique for to access the abdominal cavity is necessary about three to ten minutes.

The open laparoscopic entry is considered particularly safe in patients with previous abdominal surgery, especially midline incisions. Vascular injuries are nearly entirely prevented by the open entry technique, with anecdotal cases of aortic laceration being reported. These injuries have been attributed to an insufficient elevation of the abdominal wall, with the skin incision passing directly through skin, fascia, and into the underlying vessels [21, 23]. A factor accounting for some of this disparity could be patient selection bias. 

In literature are reported fewer injury of bowel and major vascular injury using this technique than the Verres needle technique.

A meta-analysis of 760,890 closed laparoscopy and 22,465 open laparoscopy cases reported that the incidence of vascular injury rate in closed laparoscopy was 0.44% compared with 0% in open laparoscopy. The incidence of bowel injury was 0.7% compared to 0.5%, respectively. The authors concluded that the open (Hasson) technique eliminates the risk of vascular injury and gas embolism and reduces the risk of bowel injury and recommend the open technique to be adopted for primary laparoscopic entry [24].

Penfield noted a 0.06% incidence of bowel injury, but the injuries were mostly partial and were recognised immediately because of the proximity of the bowel to the wound [25].

Patients with previous abdominal surgery and those known to have peritoneal adhesions present a higher risk for peritoneal entry complication [26]. In this case the high-risk patients must be preoperative informed of the possibility of alternate entry method or the likelihood of conversion to laparotomy. General surgeons in Canada use the open primary entry technique, with the Hasson trocar and cannula applied periumbilically to establish a pneumoperitoneum for laparoscopic surgery [27]. In a review of 2010 patients the Authors confirm previous reports of the low risk of enterotomy, absence of fatal vascular injury, and comparable rates of umbilical infection/hernia associated with an open entry technique. The rapid recognition of enterotomy with this entry technique and the utility of this technique in obese patients or those with previous abdominal procedures are additional advantages [28].

Hasson report his experience on 5,284 women who were subjected to open technique for laparoscopic surgery and have developed complications related to primary access. Twenty-one patients had minor wound infections, four had minor haematomas, one developed an umbilical hernia that required surgery, and one had an inadvertent injury to the small bowel that was repaired intraoperatively without adverse outcome [29].

A new technique emphasizes the identification and the incision of the point where the midline abdominal fascia is fused with the base of the umbilicus. The importance of the application of counter traction directly at the point of insertion has been described in literature [30]. This method allows the penetration under the direct vision with minimal controlled axial force and without the requirement of fascial sutures or other cumbersome aspects of the traditional open technique. 

Knowledge application of the anatomy of the umbilicus is critical to this method of access. The use of a lateral incision to the umbilicus allows ideal delineation of the umbilical junction with the midline fascia, and a left-sided incision is preferentially employed as this minimizes interference from the falciform ligament. The success of this method depends on identifying the single point where the umbilical fascia and the peritoneum are fused. The Incision with suture scissors of this point provides a rapid, safe, and easy access to the peritoneal cavity. This technique has been used for more than 1000 consecutive laparoscopic procedures over a 4-year period and there was only one intraabdominal injury to the small bowel in a patient with multiple previous midline laparotomies and a prosthetic mesh closure [30]. 

Another technique consists in a transverse supra- or subumbilical incision showing the umbilical cicatrix pillar and the junction of the pillar with the linea alba. After the incision (1 cm) at the junction of the umbilical cicatrix pillar with the linea alba is possible to have the peritoneal cavity opened [31]. This technique is safe, effective, easy to learn, and quick to perform. The method clearly displays the point on the abdominal wall where the peritoneum is tightly fused and allows direct entry to the peritoneal cavity in the majority of the cases, while the abdominal wall is kept tented and away from the underlying viscera at all times [31].

Probably the safest initial entry site in high-risk patients is the left upper quadrant, better known as Palmer's point. This site (3 cm below the left costal margin in the midclavicular line) is rarely affected by adhesions, and with splenomegaly and stomach distension being excluded it has been shown to be safe [32, 33]. Molloy et al. in a meta-analysis of 51 studies on techniques and complications of primary port entry, the authors found that risks associated with open entry are similar to those with direct entry. Left upper quadrant entry is available but is more complicated in obese patients and carries its own risks as well [34].

## 3. Discussion

Over the last two decades, rapid advances have made laparoscopic surgery a well-established procedure. However, because laparoscopy is relatively new, it still arouses controversy, particularly with regard to the best method for the creation of the pneumoperitoneum.

To establish the pneumoperitoneum, access to the peritoneal cavity can be gained through minilaparotomy and insertion of a laparoscopic trocar or Hasson trocar. Alternatively, an optical trocar can be blindly inserted into the peritoneal cavity, or a Verres needle may be inserted through the abdominal midline. The latter is the most frequently used technique. 

In literature are reported various cases of injury to the great vessels caused by the Verres needle. A report illustrates the difficulty in correctly diagnosing this complication, which is mainly due to the retroperitoneal position of the vessels.

Major vascular injuries caused by the insertion of the Verres needle into the abdominal midline occur even in the hands of experienced surgeons. Schäfer et al. analyzed 26 major vascular injuries and reported that only four of them (15%) had been caused by inexperienced surgeons (surgeons who had performed fewer than 50 laparoscopic procedures). The other 22 injuries (85%) had been caused either by experienced surgeons (those who had performed between 51 and 100 procedures) or very experienced surgeons (over 100 procedures performed) [35]. Thus, it is essential that the position of the needle tip after insertion be determined as accurately as possible.

In addition, analysis of intraperitoneal pressure and volume of gas insufflated at different time points during insufflation is essential to prevent gas insufflation into sites other than the peritoneal cavity. It has been established that intraperitoneal pressure levels and the total volume of gas insufflated into the peritoneal cavity at given time points can be predicted, provided that the tip of the Verres needle is in fact in the peritoneal cavity during insufflation [36]. 

No vascular injury was reported in a study investigating 3,041 patients submitted to blind insertion of the first trocar through a midline incision at the umbilicus under intraperitoneal pressure of 25–30 mmHg [37]. This corroborates the hypothesis that elevated intraperitoneal pressure protects the intraabdominal structures from injury caused by the first trocar. No injury caused by blind insertion of the first trocar was reported in a study involving 1,150 patients submitted to laparoscopy under intraperitoneal pressure of 25 mmHg [38]. No clinical complications have been shown to arise from transitory elevation of intraperitoneal pressure [37, 38].

However, it is known that extremely high levels of intraperitoneal pressure for longer periods of time can cause physiological and structural changes, directly related to the tension levels caused by the high pressure. Studies have demonstrated that patients submitted to high intraperitoneal pressure for longer periods of time can present decreased cardiac output, decreased venous return, increased mean arterial pressure, increased systemic vascular resistance, altered renal perfusion and glomerular filtration rate, and ischemia-reperfusion injury of intraabdominal organs [39]. Therefore, most authors have proposed that intraperitoneal pressure remains at 12 mmHg and never above 15 mmHg during laparoscopic procedures. 

The Verres needle is typically inserted through the abdominal midline, at the umbilicus. Albeit effective, insertion of the Verres needle through the midline poses danger. All injuries to the great vessels caused by the Verres needle reported in the literature resulted from midline punctures in the umbilical region [40]. Azevedo et al. claim that insertion of the Verres needle into the left hypochondrium has been reported as safe and effective and potential injuries are less severe [38]. Nevertheless, it is essential that the position of the needle after insertion be determined as accurately as possible. Needle-positioning tests prior to insufflation have been evaluated and considered adequate to guide surgeons with regard to the correct positioning of the Veress needle for creation of the pneumoperitoneum [41].

The objective of the study of Azevedo was to evaluate five tests that are used to confirm the correct position of the Verres needle inside the peritoneal cavity. The tests were (1) aspiration test: aspiration using a 5 mL syringe with a Verres needle. This test was considered positive when no material was aspirated and negative when material was aspirated; (2) injection test: injection of 5 mL of saline solution through the Verres needle. This test was considered positive when moderate resistance to liquid flow was observed and negative when increased resistance to liquid flow was observed; (3) recovery test: after injection of 5 mL of saline solution, aspiration was performed, this test was considered positive when the liquid injected was not recovered and negative when the liquid was not recovered; (4) saline drop test: saline solution was poured into the needle. Liquid flow was observed. This test was considered positive when the liquid disappeared immediately and negative when the liquid remained inside the needle; (5) initial intraperitoneal pressure test. This test was considered positive (needle correctly positioned inside the peritoneal cavity with unobstructed side hole) when initial intraperitoneal pressure was 8 mmHg or lower during the first ten seconds of insufflation. When initial intraperitoneal pressure was over 8 mmHg and remained this way for ten seconds, test was considered negative (needle incorrectly positioned inside the peritoneal cavity or obstruction of its side hole).

The five tests evaluated in this study are adequate to guide surgeons with regard to the correct positioning of the Verres needle for creation of pneumoperitoneum. These tests may avoid iatrogenic injury and insufflation of gas into the wrong site [41].

Although these tests and techniques may be helpful in accessing the peritoneal cavity, the fact that visceral and vascular injuries occur shows that they are not foolproof. 

A study reported that complication rates during introduction of Verres needle are one attempt 0.8–16.3%, two attempts 16.31–37.5%, three attempts 44.4–64%, and more than three attempts 84.6–100%. The complications associated were extraperitoneal insufflation, omental and bowel injuries, and failed laparoscopy [42].

Merlin et al. reported on a systematic review that the most common of the major complications associated with access were bowel injuries [43]. The risk of bowel injury in nonrandomized studies was higher with the open technique than with closed technique, although bias introduced through patient selection may have been a factor. The evidence on the comparative safety and effectiveness of the different access methods was not definitive, but trends in the data merit further exploration. 

Chapron et al. reported on a nonrandomized comparison of open versus closed laparoscopic entry practised by university affiliated hospital teams. The bowel and major vessel injury rates were 0.04% and 0.01% in the closed technique and 0.19% and 0% in the open technique, respectively. They concluded that open laparoscopy does not reduce the risk of major complications during laparoscopic access [44].

Catarci et al. analysed a multicenter questionnaire survey of general surgeons (57% responding) reported a relatively high incidence of major injuries; the highest with optical trocars (0.27%), the second highest with the closed technique (0.18%, used 82% of the time), and the lowest with the open technique (0.09%). Until 1997, no case of major retroperitoneal vessel injury had been reported with the use of a blunt Hasson's cannula, which therefore was considered to be absolutely safe, while the rate of vascular injury was from 0.02% to 0.24% for closed technique. The rate of visceral injury with closed technique varied from 0.03% to 0.15% with prevalence of injury to the gastrointestinal tract (80%) greater than that for urinary tract (20%). With the open technique, the same figure varied from 0% to 0.12%. High rates of morality related to major injury (10–50%) actually were reported in gynaecologic series, associated mainly with delayed diagnosis and treatment [45].

Jansen et al. in clinical trials that compared closed and open entry techniques, the complication rates were 0.07% and 0.17% for the closed and open techniques, respectively. The number of entry-related complications with the open technique was significantly higher than with the closed technique. Hasson et al. [5] conclude “There is no evidence to support abandoning the closed entry technique in laparoscopy; however, the selection of patients for an open or alternative procedure is still recommended” [46].

Meta-analysis failed to reveal any safety advantage of an open technique when compared with a closed method of entry, in terms of both visceral and major vascular injury. It must be noted that the included randomised controlled trials had insufficient power to effectively demonstrate an advantage [47].

The rate of carbon dioxide embolism was 0.001% in a review of 489 335 closed laparoscopies [48]. Several case reports have detailed fatal or near-fatal coronary, cerebral, or other gas embolism. Such a complication has not been reported at open laparoscopy.

Tinelli et al. present a modified direct optical entry (DOE) in patients previously undergone abdominal pelvic surgery, compared with the classical open laparoscopy. The authors suggest that DOE is as safe as open laparoscopy and can be used in patients with previous abdomino-pelvic surgery [49].

The use of Hasson technique has not cost due to the complete surgical procedure do not need any disposable device. On the opposite Verres needle has the cost of the Verres disposable device permitting only one use. The cost of complications varies depending on the type of complication. The major complications are vascular injury and bowel injury. The complete amount of the complication depends from the operating room cost, the hospitalization duration cost, and from the supplementary devices utilized for the complication. The cost of operating room per hour is about 300€, while the cost of hospitalization is estimated at about 600€ a day. In case of vascular injury the possible use of prosthesis must be added, while in the case of intestinal injury the possible use of the stapler should be added.

In our personal experience based on 750 laparoscopic surgical procedure, 50 patients were previously undergone to abdominal surgery. Between these patients only one reported a minor intestinal injury during Verres procedure repaired during the laparoscopic procedure injury.

In conclusion between two techniques was analysed the Hasson technique that could be preferred in case of operated patients without comorbidities.
